# Influence of co-morbid fibromyalgia on disease activity measures and response to tumour necrosis factor inhibitors in axial spondyloarthritis: results from a UK national register

**DOI:** 10.1093/rheumatology/key206

**Published:** 2018-07-20

**Authors:** Gary J Macfarlane, Ross I R MacDonald, Ejaz Pathan, Stefan Siebert, Karl Gaffney, Ernest Choy, Jon Packham, Kathryn R Martin, Kirstie Haywood, Raj Sengupta, Fabiola Atzeni, Gareth T Jones

**Affiliations:** 1 Epidemiology Group, School of Medicine, Medical Sciences and Nutrition, Aberdeen, UK; 2 Aberdeen Centre for Arthritis and Musculoskeletal Health, University of Aberdeen, Aberdeen, UK; 3 Department of Rheumatology, Aberdeen Royal Infirmary, Aberdeen, UK; 4 Institute of Infection, Immunity and Inflammation, University of Glasgow, Glasgow, UK; 5 Department of Rheumatology, Norfolk & Norwich University Hospitals NHS Foundation Trust, Norwich, UK; 6 Division of Infection and Immunity, Cardiff University School of Medicine, Cardiff, UK; 7 Research Institute for Applied Clinical Sciences, Keele University, Keele, UK; 8 Warwick Research in Nursing, Division of Health Sciences, Warwick Medical School, University of Warwick, Coventry, UK; 9 Department of Rheumatology, Royal National Hospital for Rheumatic Diseases NHS Foundation Trust, Bath, UK; 10 IRCCS Galeazzi Orthopaedic Institute, Milan, Italy

**Keywords:** axial spondyloarthritis, biologic therapy, cohort study, co-morbidity, disease activity, disease register, epidemiology, fibromyalgia, outcome, response

## Abstract

**Objective:**

To quantify the extent to which co-morbid FM is associated with higher disease activity, worse quality of life (QoL) and poorer response to TNF inhibitors (TNFis) in patients with axial SpA.

**Methods:**

A prospective study recruiting across 83 centres in the UK. Clinical information and patient-reported measures were available, including 2011 criteria for FM. Multivariable linear regression was used to model the effect of meeting the FM criteria on disease activity, QoL and response to TNFis.

**Results:**

A total of 1757 participants were eligible for analyses, of whom 22.1% met criteria for FM. Those with co-morbid FM criteria had higher disease activity [BASDAI average difference FM^+^ − FM^−^ 1.04 (95% CI 0.75, 1.33)] and worse QoL [Ankylosing Spondylitis Quality of Life score difference 1.42 (95% CI 0.88, 1.96)] after adjusting for demographic, clinical and lifestyle factors. Among 291 participants who commenced biologic therapy, BASDAI scores in those with co-morbid FM were 2.0 higher at baseline but decreased to 1.1 higher at 12 months. There was no significant difference in the likelihood of meeting Assessment of SpondyloArthritis international Society 20 criteria at 12 months. Less improvement in disease activity and QoL over 3 months of TNFi therapy was most strongly related to high scores on the FM criteria symptom severity scale component.

**Conclusion:**

Fulfilling criteria for FM has a modest impact on the assessment of axial SpA disease activity and QoL and does not significantly influence response to biologic therapy. Those with a high symptom severity scale on FM assessment may benefit from additional specific management for FM.

**Rheumatology key messages**

- One in five patients recruited to the BSR register for patients with axial SpA meet research criteria for FM.
- Axial SpA patients with co-morbid FM were equally likely to meet response criteria at 12 months.
- Higher scores on the FM criteria symptom severity scale predicted less benefit from TNF inhibitors.

## Introduction

The issue of FM as a co-morbidity to axial SpA (axSpA) is of considerable recent interest. In July 2013, the US Food and Drug Administration (FDA) met to consider TNF inhibitors (TNFis) in patients with non-radiographic axSpA based on the Assessment of SpondyloArthritis international Society (ASAS) classification criteria [1]. The FDA Arthritis Advisory Committee recognized the unmet need for effective pharmacologic therapy for patients who had positive MRI rather than radiographic changes, or based on positive HLA-B27 plus other characteristic SpA features, but who did not fulfil the modified New York (mNY) criteria for AS [2]. However, they were concerned about the specificity of the ASAS criteria [3] and the possibility that patients with highly prevalent conditions such as mechanical back pain or FM might be incorrectly diagnosed with non-radiographic axSpA and be inappropriately treated with TNFi medications. This highlights the need to better understand the characteristics of axSpA patients who have co-morbid FM in order to assess and distinguish the two conditions (including when they coexist) and to develop treatment strategies that can effectively work in parallel.

This led to research that sought to understand how often axSpA and FM co-occur. Notwithstanding the fact that research criteria for FM have not been validated in the context of inflammatory rheumatic conditions, studies have sought to understand how often people with axSpA met one or more of the criteria for FM. These demonstrated that co-occurrence was common. We have shown that 21% of 1504 persons within the British Society for Rheumatology Biologics Register of AS (BSRBR-AS) met 2011 criteria for FM (also known as the modified 2010 criteria and as research criteria) [4]. In a smaller study of 200 patients meeting ASAS criteria for axSpA, Baraliakos *et al.* [5] found that 24% met the above research criteria while 14% met the previous 1990 ACR criteria. This is consistent with the observation of high prevalence of FM in inflammatory rheumatic diseases generally [6]. However, identifying co-morbid FM in people with axSpA is challenging. The ACR 1990 criteria for FM require the report of axial skeleton pain, which is one of the key clinical features of axSpA. These criteria, as well as the 2011 criteria, require multisite pain, which is also reported by axSpA patients due to inflammatory enthesitis/synovitis [7, 8].

The key issue is distinguishing and providing appropriate management for both conditions when they occur together. A pooled analysis of data from clinical trials treating axSpA patients with etanercept, SSZ or placebo showed a higher disease burden and poorer response to treatment in women and identified the possibility that this may be due to concomitant FM [9, 10]. We currently do not know how patients with co-morbid FM respond to TNFi therapy compared with those without. However, several standard disease indices, including the BASDAI, as well as wider measures of disease impact [such as the Ankylosing Spondylitis Quality of Life (ASQoL) index] are based entirely on patient reports and may be inflated due to co-morbid FM. This could lead to inappropriate management since guidelines include BASDAI score as one determinant for use of TNFi therapy [11–13].

The purpose of this analysis is therefore 2-fold: to quantify the extent to which meeting criteria for FM is associated with higher measures of disease activity and impact (aim 1) and to determine whether meeting research criteria for FM is associated with poorer response on first use of TNFi therapy (aim 2).

## Methods

The BSRBR-AS is a prospective cohort study that has recruited patients from 83 secondary care centres in the UK who have a physician diagnosis of axSpA and meet the ASAS defined criteria. Recruitment started in December 2012, initially for people meeting the ASAS imaging criteria for axSpA. Patients meeting only ASAS clinical criteria were subsequently eligible to be recruited in November 2014. All participants are naïve to TNFi therapy at the time of recruitment but may either be starting such therapy or continuing on current non-TNFi therapy. The study protocol has previously been published [14] but, in brief, participants starting TNFi therapy have clinical and patient-reported information collected at the start of therapy and 3, 6 and 12 months later. Those not on TNFi therapy have information collected at recruitment and annually thereafter but may transfer to the follow-up schedule of participants on TNFi therapy if they commenced such therapy during the course of the study. Eligible TNFi therapies were adalimumab, etanercept and certolizumab pegol. From September 2015, the patient-reported data included the 2011 FM criteria.

Data collected from or measured on each participant at recruitment and each follow-up point included cigarette smoking (current, ex-smoker, never smoker); the BASDAI, BASFI and BASMI [15–17]; the 18-item ASQoL scale, providing a score from 0 [good quality of life (QoL)] to 18 (poor QoL) [18] and the Hospital Anxiety and Depression Scale (HADS), a measure of emotional distress, anxiety disorders and depression. There are two subscales, for anxiety and depression, each with scores ranging from 0 to 21, with higher scores indicating more severe problems [19].

Information was collected in relation to the 2011 FM criteria [8]. There are two components to the criteria: the Widespread Pain Index (WPI) and the Symptom Severity Scale (SSS). The WPI records in how many of 19 body areas the respondent reports pain in the past week (score 0–19). For the SSS, respondents indicate the severity of fatigue, waking unrefreshed and cognitive symptoms such as brain fog over the past week (scored 0–3 each). The criteria also include three items on whether depression, headaches and pain or cramps in the lower abdomen have occurred in the past 6 months (score 1 each if present), giving a maximum total score of 12.

CRP was measured at recruitment but was only measured subsequently if clinically indicated. A measure of socio-economic status, the Index of Multiple Deprivation (IMD), was derived from the postcode of the residence of participants and categorized into quintiles with references to their country of residence [20, 21].

Ethical approval was obtained from the National Research Ethics Service Committee North East—County Durham and Tees Valley (reference 11/NE/0374) and informed consent was obtained from all participants.

### Analysis

#### Aim 1

Participants were included if they had completed the FM criteria either at recruitment or follow-up. Data from the first completion of the items that contribute to this criteria were used (and are referred to as baseline). The effect of FM status on baseline BASDAI and ASQoL was determined. Thereafter, multivariate linear regression analyses were used to evaluate the influence of FM status on baseline disease activity (BASDAI) adjusted for BASMI and CRP (both measured within 3 months of the self-report data), BASFI, age group, gender, IMD, disease management (on a TNFi) and smoking status and baseline ASQoL adjusted for BASDAI, BASFI, BASMI, age group, gender, IMD, disease management and smoking status. As the availability of CRP restricted the numbers available for analysis, and it was shown not to be related to BASDAI, it was only included in an additional (sensitivity analysis) model predicting ASQoL. Both the BASDAI and ASQoL analyses were first conducted with a dichotomous FM status variable and then using the WPI and SSS components of the criteria instead.

#### Aim 2

Participants were included in this analysis if they had completed FM research criteria within the 6 months before or 1 month after commencing TNFi therapy for the first time. They were also required to have completed at least one follow-up questionnaire 3, 6 or 12 months later. Two-sample *t*-tests were used to compare differences in BASDAI and ASQoL between patients meeting FM criteria (called FM^+^) and those who did not (FM^−^) at baseline and 3, 6 and 12 months, as well as ASAS20 and ASAS40 responses at each of these follow-up points. In predicting the contribution of FM status on the change in BASDAI after 3 months, adjustment was made for baseline BASDAI, BASFI, age group, IMD, gender and smoking status, while in the analysis predicting ASQoL change after 3 months, adjustment was made additionally for baseline ASQoL. Analysis was again conducted first with dichotomous FM criteria status and then with the WPI and SSS components of the criteria. Inclusion of clinically measured variables reduced the sample size available to the analysis, but a sensitivity analysis with CRP and BASMI was included to investigate their effects. We separately included baseline HADS to determine whether this mediated the relationship between FM status and treatment response. All analyses were conducted using Stata 14 SE 64-bit (StataCorp, College Station, TX, USA) for statistical analysis and the June 2017 study dataset.

## Results

A total of 1757 participants (67% male) completed the research criteria for FM on at least one occasion and were eligible for the current analyses. Their median age was 50.8 years, with a median time since symptom onset of 27 years, and 80.2% of those who had been tested were HLA-B27 positive. Most participants (66.8%) met the mNY criteria for AS, an additional 28.4% fulfilled ASAS imaging criteria but not mNY and 4.8% fulfilled only ASAS clinical criteria for axSpA.

### Influence of FM status on disease activity and QoL (aim 1)

Those who were FM^+^ at baseline [*n* = 388 (22.1%)] had higher BASDAI scores than those who were FM^−^ [6.7 *vs* 3.6; difference 3.1 (95% CI 2.8, 3.3)]. A higher BASDAI score was independently predicted by being FM^+^ (1.04 higher average scores) in a multivariable linear regression model (which included participants who had a CRP within 3 months of the self-reported information; *n* = 1093) (Table 1). Additional predictors were higher BASFI (0.67 average increase in BASDAI per unit increase in BASFI), lower BASMI (0.14/U), younger age group and not being on a TNFi (0.34 higher average score). BASDAI was not significantly related to CRP, gender, smoking or IMD. When the individual component scores of the FM criteria were entered in the model (instead of the dichotomous FM variable), BASDAI was related to both the WPI score (0.11 average increase in BASDAI for every additional area of pain reported) and the SSS (0.20 average increase/unit).

**Table 1:** Predictors of the BASDAI score at baseline

Baseline variable	Model 1 (*n* = 705), coefficient (95% CI)	Model 2 (*n* = 626), coefficient (95% CI)
Constant	2.54 ( 1.97, 3.12)	1.33 (0.73, 1.93)
BASMI	−0.14 (−0.22, −0.07)	−0.08 (−0.15, −0.00)
BASFI	0.67 (0.62, 0.73)	0.51 (0.45, 0.57)
CRP, mg/dl	−0.00 (−0.01, 0.01)	−0.00 (−0.01, 0.01)
Age, years		
<30	0	0
30–39	−0.26 (−0.75, 0.22)	−0.16 (−0.62, 0.30)
40–49	−0.41 (−0.89, 0.07)	−0.15 (−0.61, 0.30)
50–59	−0.50 (−0.98, −0.01)	−0.28 (−0.75, 0.18)
60–69	−0.86 (−1.40, −0.33)	−0.47 (−0.98, 0.04)
≥70	−1.03 (−1.62, −0.45)	−0.58 (−1.15, 0.00)
Gender		
Male	0	0
Female	0.20 (−0.04, 0.43)	0.06 (−0.17, 0.30)
Deprivation (quintiles)		
1 (highest deprivation)	0	0
2	−0.16 (−0.57, 0.24)	−0.12 (−0.51, 0.28)
3	−0.33 (−0.73, 0.06)	−0.31 (−0.70, 0.09)
4	−0.11 (−0.49, 0.27)	−0.10 (−0.48, 0.28)
5	−0.33 (−0.73, 0.06)	−0.25 (−0.64, 0.15)
Management		
Biologic	−0.34 (−0.58, −0.09)	(−0.53, −0.06)
Smoking status		
Never	0	0
Ex	0.04 (−0.21, 0.28)	−0.01 (−0.24, 0.23)
Current	0.01 (−0.31, 0.33)	−0.01 (−0.32, 0.31)
FM	1.04 ( 0.75, 1.33)	Not entered
FM	Not entered	
WPI		0.11 (0.08, 0.15)
SSS		0.20 (0.15, 0.25)

Model 1 fit: *R*^2^ = 0.6454; model 2 fit: *R*^2^ = 0.7055.

Those who were FM^+^ at baseline had poorer QoL scores than those who were FM^−^ [13.1 *vs* 6.1; difference 7.0 (95% CI 6.5, 7.6)]. Poorer QoL at baseline was predicted, on multivariable analysis, by being FM^+^ (1.42 higher average ASQoL) in addition to higher BASDAI score (0.85 increase in ASQoL per unit increase in BASDAI), higher BASFI (1.00/U), lower BASMI (0.13/U), female gender (0.74 higher average ASQoL score) and being a current smoker (0.94 higher average score) (Table 2). QoL increased with older age group but was not related to TNFi management or IMD. When the FM component scores were entered, poorer QoL was strongly related to SSS (0.50 increase in ASQoL/unit), with a 0.09 in increase in ASQoL per unit increase in WPI. As a sensitivity analysis, when the CRP was included in model 2 it was not related to QoL [coefficient 0.00 (95% CI −0.02, 0.02)].

**Table 2:** Predictors of the ASQoL score at baseline

Baseline variable	Model 1 (*n* = 886), coefficient (95% CI)	Model 2 (*n* = 796), coefficient (95% CI)
Constant	0.88 (−0.17, 1.93)	−0.88 (−1.94, 0.18)
BASDAI	0.85 ( 0.72, 0.99)	0.54 ( 0.39, 0.68)
BASFI	1.00 ( 0.87, 1.13)	0.91 ( 0.78, 1.04)
BASMI	−0.13 (−0.26, −0.00)	−0.10 (−0.23, 0.03)
Age (years)		
<30	0	0
30–39	−0.34 (−1.18, 0.50)	−0.05 (−0.85, 0.76)
40–49	−1.10 (−1.93, −0.28)	−0.64 (−1.43, 0.15)
50–59	−1.55 (−2.40, −0.71)	−1.07 (−1.88, −0.25)
60–69	−1.71 (−2.63, −0.79)	−0.76 (−1.66, 0.13)
≥70	−2.20 (−3.21, −1.19)	−1.31 (−2.31, −0.31)
Gender		
Male	0	0
Female	0.74 (0.33, 1.16)	0.58 ( 0.16, 0.99)
Index of Multiple Deprivation (quintiles)		
1 (highest deprivation)	0	0
2	−0.14 (−0.85, 0.57)	0.03 (−0.68, 0.74)
3	−0.14 (−0.84, 0.55)	0.11 (−0.60, 0.82)
4	−0.24 (−0.90, 0.42)	−0.09 (−0.76, 0.58)
5	−0.34 (−1.02, 0.34)	−0.15 (−0.84, 0.55)
Management		
Biologic therapy	0.11 (−0.33, 0.56)	−0.01 (−0.45, 0.43)
Smoking status		
Never	0	0
Ex-smoker	0.05 (−0.37, 0.47)	0.05 (−0.36, 0.46)
Current	0.94 ( 0.38, 1.49)	0.97 ( 0.42, 1.52)
FM	1.42 ( 0.88, 1.96)	Not entered
FM	Not entered	
WPI		0.09 (0.02, 0.16)
SSS		0.50 (0.41, 0.59)

Model 1 fit: *R*^2^ = 0.7467; model 2 fit: *R*^2^ = 0.7821.

### Response to TNFi therapy according to FM status (aim 2)

**Fig. 1:**
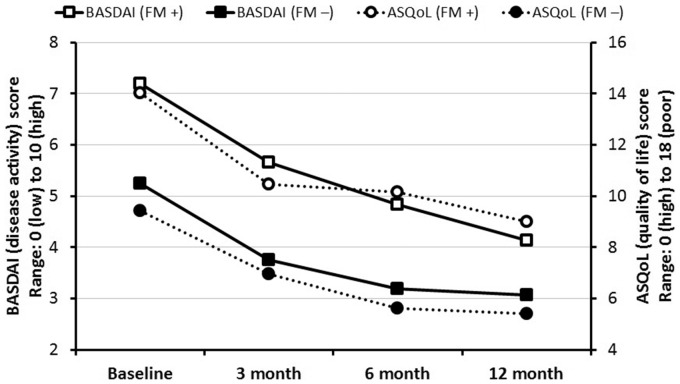
Disease activity and QoL after commencement of biologic therapy

There were a total of 291 participants who commenced TNFi therapy and had completed FM criteria within the required timescale. Of these, 139, 123 and 74 had reached the follow-up and completed a questionnaire 3, 6 and 12 months later, respectively. At the time of commencing TNFi therapy, participants who were FM^+^ had significantly higher BASDAI scores [7.2 *vs* 5.2; difference 2.0 (95% CI 1.5, 2.4)]. They continued to have higher scores throughout the follow-up, although the magnitude of the difference decreased over time: 3 months [5.7 *vs* 3.7; difference 1.9 (95% CI 1.0, 2.8)], 6 months [4.8 *vs* 3.2; difference 1.6 (95% CI 0.7, 2.6)] and 12 months [4.1 *vs* 3.1; difference 1.1 (95% CI −0.0, 2.2)]. QoL was poorer among those who were FM^+^ [14.0 *vs* 9.4; difference 4.6 (95% CI 3.5, 5.7)] and remained so at 3 months [10.5 *vs* 7.0; difference 3.5 (95% CI 1.5, 5.5)], 6 months [10.2 *vs* 5.6; difference 4.6 (95% CI 2.5, 6.6)] and 12 months [9.0 *vs* 5.4; difference 3.6 (95% CI 0.9, 6.3)] (Fig. 1). It is notable in FM^+^ patients that BASDAI continues to decrease throughout the 12 month follow-up period. Throughout the follow-up, those originally FM^+^ were less likely to meet ASAS20 response criteria at all time points. The differences decreased throughout the follow-up and none were statistically significant: 3 months [36% *vs* 46%; difference −10% (95% CI −28, 8)], 6 months [56 *vs* 61%; difference −5% (95% CI −24, 14)] and 12 months [60 *vs* 63%; difference −4% (95% CI −30, 23)]. Similar-size differences in response were observed for ASAS40: 3 months [24 *vs* 34%; difference −11% (95% CI −28, 7)], 6 months [39 *vs* 44%; difference −5% (95% CI −24, 14)] and 12 months [32 *vs* 42%; difference −11% (95% CI −37, 16)]. The proportion of participants who were FM^+^ at baseline and who continued to meet the criteria at follow-up was 36.2% at 3 months, 40.5% at 6 months and 40% at 12 months. The decrease in the proportion of patients fulfilling the FM criteria over time was due to improvements in both WPI and SSS. WPI improved by 1.5, 1.8 and 1.4 over 3, 6 and 12 months, respectively, and SSS improved by 0.8, 1.2 and 0.8, respectively. These represent very similar improvements as a percentage of the relevant maximum scale score (e.g. 8 and 7% at 3 months for WPI and SSS, respectively).

A multivariable model predicting the change in BASDAI (BASDAI_baseline_ – BASDAI_3 months_) demonstrated that those who were FM^+^ at baseline had 0.58 less improvement in BASDAI than those who were FM^−^, but this was not statistically significant (Table 3). Larger improvements were related to higher baseline BASDAI (every unit increase in BASDAI associated with an average 0.72 greater improvement in BASDAI) and lower baseline BASFI (0.38 less improvement/unit increase). However, when the effect of the individual components of FM criteria were considered, higher scores on the SSS were significantly associated with a poorer response (0.32 lower average improvement per unit increase in the SSS). When CRP or BASMI was added to model 2 (as a sensitivity analysis, since their inclusion restricted numbers available for analysis), they were not associated with an improvement in BASDAI [0.00 (95% CI −0.02, 0.03) and 0.21 (95% CI −0.06, 0.48), respectively] and neither was HADS (anxiety) [severe anxiety 0.18 (95% CI −1.36, 1.72) per unit increase in score] or HADS (depression) [severe depression −0.51 (95% CI −2.45, 1.42) per unit increase in score] when put into the model together.

**Table 3:** Predicting response to biologic therapy: improvements in the BASDAI

Baseline variable	Model 1 (*n* = 135), coefficient (95% CI)	Model 2 (*n* = 121), coefficient (95% CI)
Constant	−0.99 (−2.72, 0.75)	−0.28 (−2.03, 1.48)
BASDAI	0.72 (0.49, 0.95)	0.84 (0.60, 1.08)
BASFI	−0.38 (−0.60, −0.17)	−0.17 (−0.41, 0.07)
Age (years)		
<30	0	0
30–39	0.75 (−0.56, 2.07)	0.82 (−0.46, 2.10)
40–49	0.58 (−0.73, 1.89)	0.29 (−0.98, 1.56)
50–59	0.31 (−1.11, 1.73)	0.26 (−1.14, 1.66)
60–69	0.41 (−1.03, 1.86)	0.13 (−1.35, 1.62)
≥70	−1.03 (−3.25, 1.19)	−0.89 (−3.22, 1.43)
Index of Multiple Deprivation (quintiles)		
1 (highest deprivation)	0	0
2	0.58 (−0.71, 1.87)	0.66 (−0.68, 2.00)
3	−0.20 (−1.43, 1.03)	−0.50 (−1.79, 0.80)
4	0.91 (−0.28, 2.11)	0.71 (−0.52, 1.94)
5	0.55 (−0.67, 1.78)	0.19 (−1.12, 1.49)
Gender		
Male	0	0
Female	−0.61 (−1.38, 0.17)	−0.10 (−0.91, 0.70)
Smoking status		
Never	0	0
Ex-smoker	0.13 (−0.72, 0.98)	0.21 (−0.64, 1.06)
Current	0.19 (−0.76, 1.13)	0.59 (−0.40, 1.57)
FM criteria met	−0.58 (−1.40, 0.23)	Not applicable
FM	Not applicable	
WPI		−0.10 (−0.24, 0.03)
SSS		−0.32 (−0.53, −0.12)

Model 1 fit: *R*^2^ = 0.3261; model 2 fit: *R*^2^ = 0.4079.

A corresponding analysis was run with QoL as the outcome (ASQoL_baseline_ – ASQoL_3 months_). High scores on the SSS for the FM criteria were predictive of lower improvement in QoL, as were poorer QoL and worse disease activity on commencing treatment (Table 4). When CRP or BASMI was added to model 2 (again as a sensitivity analysis), they were not associated with improvement in ASQoL [0.11 (95% CI −0.47, 0.69) and −0.01 (95% CI −0.07, 0.06), respectively] and neither was HADS (anxiety) [severe anxiety −0.79 (95% CI −4.13, 2.55)] or HADS (depression) [severe depression −3.29 (95% CI −7.48, 0.91)].

**Table 4:** Predicting response to biologic therapy: improvements in QoL (ASQoL score)

Variable	Model 1 (*n* = 133), coefficient (95% CI)	Model 2 (*n* = 119), coefficient (95% CI)
Constant	−0.93 (−4.72, 2.86)	−0.15 (−3.96, 3.66)
ASQOL	0.30 (0.01, 0.59)	0.52 (0.20, 0.84)
BASDAI	0.36 (−0.17, 0.89)	0.52 (−0.03, 1.06)
BASFI	−0.50 (−1.06, 0.06)	−0.23 (−0.82, 0.35)
Age (years)		
30–39	2.37 (−0.46, 5.21)	2.31 (−0.46, 5.08)
0–49	2.16 (−0.71, 5.03)	1.75 (−1.03, 4.53)
50–59	0.93 (−2.20, 4.07)	1.01 (−2.07, 4.09)
60–69	0.90 (−2.30, 4.10)	0.49 (−2.74, 3.72)
≥70	0.82 (−3.99, 5.62)	1.60 (−3.42, 6.63)
Index of Multiple Deprivation (quintiles)		
1 (highest deprivation)	0	0
2	−1.27 (−4.05, 1.51)	−0.45 (−3.35, 2.46)
3	−1.28 (−3.97, 1.42)	−1.01 (−3.85, 1.83)
4	0.77 (−1.80, 3.35)	0.95 (−1.71, 3.61)
5	−0.40 (−3.04, 2.24)	−0.61 (−3.42, 2.20)
Gender		
Female	−0.67 (−2.37, 1.04)	0.17 (−1.60, 1.94)
Smoking status		
Never	0	0
Ex-smoker	1.31 (−0.55, 3.17)	1.33 (−0.51, 3.18)
Current	0.43 (−1.72, 2.57)	0.72 (−1.48, 2.92)
FM criteria met	−0.51 (−2.29, 1.26)	Not applicable
FM	Not applicable	
WPI		−0.19 (−0.49, 0.10)
SSS		−0.74 (−1.22, −0.25)

Model 1 fit: *R*^2^ = 0.1830; model 2 fit: *R*^2^ = 0.2896.

## Discussion

Patients with axSpA who were FM^+^ had only modestly higher disease activity and worse QoL, after adjustment for disease indices, demographic and socio-economic factors. Poor QoL was more strongly determined by a high score on the SSS of FM criteria, indicating a high burden of somatic symptoms. Persons who were FM^+^ had higher BASDAI scores on commencement of TNFi therapy and throughout the 12 month follow-up, although the difference in magnitude decreased over the period of treatment. There was no significant difference in the likelihood of meeting ASAS20 or ASAS40 response criteria according to FM status. While FM status was not significantly related to response to therapy, as assessed by BASDAI or ASQoL, high somatic symptom burden was associated with worse response. Approximately two in five persons who met FM criteria at commencement of therapy continued to do so at each follow-up over the year.

The BSRBR-AS is a national register involving non-specialist and specialist centres and thus the patients recruited are likely to represent the spectrum encountered in routine clinical practice. The study protocol dictated that participants were followed up clinically and by questionnaire at 3, 6 and 12 months. This schedule was chosen to fit in with routine clinical practice. If the routine follow-up did not occur (or sufficient time had not passed for the follow-up to be due) or the participant did not return the questionnaire, then they could not fully participate in all the analyses presented. Therefore, for the 12 month follow-up in particular, the numbers analysed are considerably lower than those recruited. However, it should be noted that the patterns of response are very similar across the follow-up and therefore this is unlikely to have impacted the interpretation of results. Specifically, we examined whether BASDAI or ASQoL were importantly or statistically significantly related to the likelihood of follow-up and confirmed they were not. Similarly, we opted not to use the ASDAS as an outcome measure because of the necessity that the clinic visit (for the CRP) and the questionnaire (for self-reported measures) occur sufficiently close in time. CRP was shown not to be related to BASDAI (at baseline) or as a predictor of response to therapy and did not play an important part in the analyses. While the patient-reported measures could be performed without a clinic visit, the BASMI required that a clinical visit had occurred. However, the BASMI was shown not to be importantly related to disease activity or a predictor of response.

In interpreting the results of this study it is important to consider that although we were able to determine whether participants met research criteria, this is not the same as a clinical diagnosis of FM. Distinguishing, for example, multisite pain of axSpA from the axial and widespread pain of FM is extremely challenging. As previously noted, the criteria for FM have not been validated in people with inflammatory arthritis and indeed the 2010 [22] and 2011 research criteria [8] (but not the most recent 2016 criteria [23]) have sought to exclude persons from meeting FM criteria if they have symptoms that could be explained by inflammatory conditions.

This is one of the first studies to examine these issues in relation to co-morbid FM in people with axSpA. We and others have previously reported that disease indices are substantially elevated in patients who meet FM criteria [4, 24]. This study provides new information that when the comparison of FM^+^ and FM^–^ patients takes account of clinical, demographic and lifestyle differences between the groups, the effect on disease indices is much less pronounced. Using the FM rapid screening tool, (FIRST) Bello *et al.* [24] found that those who scored high on the tool were more likely to discontinue TNFi therapy and that this was a predictor of discontinuation of first therapy (together with peripheral involvement) on multivariable analysis. Molto *et al.* [25] found that response to therapy was lower in those who scored high on the FIRST for most endpoints, but not CRP. This study confirms this but has looked at a longer-term outcome (12 months *vs* 3 months) and, using internationally accepted criteria, has identified one specific FM component (SSS), rather than meeting FM criteria generally, that identifies persons most likely to have a poor response.

The clinical implications from this study are that since meeting criteria for FM *per se* only had a modest effect on BASDAI (i.e. 1 point) or ASQoL (1.5 points), there should not be undue concern that FM distorts disease indices. Being FM^+^ also did not predict poor or non-response to TNFi therapy among axSpA patients. Indeed, with TNFi therapy and a reduction in BASDAI, three of five people with co-morbid FM will no longer meet the criteria for FM. Specifically, the widespread distribution of pain was not a key determinant of response, but instead it was the high somatic symptom burden captured by the SSS of the FM criteria that was a strong predictor. As an example, assuming a patient had an SSS of 12 and a WPI of 2, then the predicted improvement on BASDAI would be 4 less than a patient scoring zero on both scales, whereas a patient with an SSS of 2 and WPI of 14 would have an improvement only 2 less than a patient scoring zero on both scales. Specifically we did not find that mood was an independent predictor of response. For patients with a high SSS, treatments employing a cognitive behaviour approach, which have been shown to be effective for FM [26] may be indicated, and studies to test the feasibility of such an approach are under way.

In summary, meeting the criteria for FM in this study had only a modest impact on the assessment of disease activity by the BASDAI and did not influence the response to TNFi therapy. A high score on the SSS, representing a high somatic symptom burden, was a greater influence on QoL, assessed by ASQoL and identified persons who had significantly poorer response to TNFi therapy. It may be useful for rheumatologists to identify patients with a high SSS who are commencing TNFi therapy and to consider additional non-pharmacological therapies to target such symptoms and potentially improve outcome.
